# Changes in Forest Soil Properties in Different Successional Stages in Lower Tropical China

**DOI:** 10.1371/journal.pone.0081359

**Published:** 2013-11-14

**Authors:** Yuelin Li, Fangfang Yang, Yangxu Ou, Deqiang Zhang, Juxiu Liu, Guowei Chu, Yaru Zhang, Dennis Otieno, Guoyi Zhou

**Affiliations:** 1 Key Laboratory of Vegetation Restoration and Management of Degraded Ecosystems, South China Botanical Garden, Chinese Academy of Sciences, Guangzhou, China; 2 University of Chinese Academy of Sciences, Beijing, China; 3 Department of Plant Ecology, University of Bayreuth, Bayreuth, Germany; DOE Pacific Northwest National Laboratory, United States of America

## Abstract

**Background:**

Natural forest succession often affects soil physical and chemical properties. Selected physical and chemical soil properties were studied in an old-growth forest across a forest successional series in Dinghushan Nature Reserve, Southern China.

**Methodology/Principal Findings:**

The aim was to assess the effects of forest succession change on soil properties. Soil samples (0–20 cm depth) were collected from three forest types at different succession stages, namely pine (*Pinus massoniana*) forest (PMF), mixed pine and broadleaf forest (PBMF) and monsoon evergreen broadleaf forest (MEBF), representing early, middle and advanced successional stages respectively. The soil samples were analyzed for soil water storage (SWS), soil organic matter (SOM), soil microbial biomass carbon (SMBC), pH, NH_4_^+^-N, available potassium (K), available phosphorus (P) and microelements (available copper (Cu), available zinc (Zn), available iron (Fe) and available boron (B)) between 1999 and 2009. The results showed that SWS, SOM, SMBC, Cu, Zn, Fe and B concentrations were higher in the advanced successional stage (MEBF stage). Conversely, P and pH were lower in the MEBF but higher in the PMF (early successional stage). pH, NH_4_^+^-N, P and K declined while SOM, Zn, Cu, Fe and B increased with increasing forest age. Soil pH was lower than 4.5 in the three forest types, indicating that the surface soil was acidic, a stable trend in Dinghushan.

**Conclusion/Significance:**

These findings demonstrated significant impacts of natural succession in an old-growth forest on the surface soil nutrient properties and organic matter. Changes in soil properties along the forest succession gradient may be a useful index for evaluating the successional stages of the subtropical forests. We caution that our inferences are drawn from a pseudo-replicated chronosequence, as true replicates were difficult to find. Further studies are needed to draw rigorous conclusions regarding on nutrient dynamics in different successional stages of forest.

## Introduction

Vegetation and soil are the two main components of terrestrial ecosystems and the succession of vegetation is often accompanied by changes in soil properties [1-4]. With reference to the interactive soil-vegetation feedback, soil provides essential nutrients for vegetation growth and development, and this in turn may drive some of the changes in soil formation and modification [5,6]. 

Soil properties are considered one of the major factors that affect the distribution patterns of forest types [7]. Soil properties from each preceding community, which increase in organic matter and profile development, together with modiﬁcation of the light environment, are the two main explanations for succession [8]. Successional vegetation plays an essential role in making nutrients available from the soil's total reserve [3]. A study in evergreen broad-leaved forests in Eastern China showed that soil total N and P increased with forest succession [9]. The impacts of vegetation on soil can also be illustrated by land degradation, which is a regressive succession. Several studies showed that land degradation is accompanied with biotic changes of diversity loss, chemical changes of decreasing soil nutrients, and physical changes with respect to infiltration, percolation, aeration, and, ultimately, erodability [4,10,11].

Understanding the relationship between plant community succession stages and soil nutrient dynamics is crucial for predicting the future of forests in light of climate change, for the sustainable management as well as for rehabilitation of soils [12]. Furthermore, soil is usually affected by management practices such as cultivation, harvesting and burning, so understanding soil biogeochemistry is essential to the stewardship of ecosystem services provided by soils. Hence, research of vegetation features and soil environment has become a popular field in ecological research, and has mainly focused on the changes in soil properties [2-4], assessment of soil fertility [13-15], and the determination of the relationship between soil nutrients and vegetation distribution [16,17] during different successional stages.

Dinghushan Natural Forest Reserve in Guangdong Province, China, is a preserved natural monsoon evergreen broadleaved forest in the subtropical zone. This forest has a history of more than 400 years with different forest communities at varying succession stages [18,19], although disturbances do exist. Long term studies show the monsoon evergreen forest in southeast China is playing an important role as a carbon sink [12,19], suggesting that old forests can also accumulate carbon in soils despite a late stage of succession. Other important questions raised by the long term study include the fate of other nutrients like N, P, and soil organic matter (SOM) along the successional process and whether these nutrients have the same trend as carbon. Current studies have shown that there are changes in available soil nutrients during the process of forest succession [12,20]. Despite these studies on nutrient dynamics in different types of forest, there is still insufficient research to reasonably predict the effects of forest succession on soil nutrients. 

Our research aims to consider the variation of several soil nutrient elements under forest succession. We therefore hypothesize that most soil nutrients increase during forest succession. Based on soil monitoring data from 1999 to 2009, the objectives of this study were to 1) characterize the fundamental physiochemical properties of forest soils under different succession stages and 2) identify the relationship between these properties and the forest succession stages in the subtropical monsoon forests in Southern China. 

## Materials and Methods

### Ethics statement

The study site is maintained by South China Botanical Garden, Chinese Academy of Sciences. The location is within the Dinghushan Natural Forest Reserve, Guangdong Province, Southern China. All necessary permits were obtained for the described field study. The field study did not involve endangered or protected species. Data will be made available upon request.

### Site description

The study was conducted in Dinghushan Natural Forest Reserve (23°09′N, 112°33′E), Guangdong Province, Southern China. The forest covers 1156 ha, with an elevation range from 14 m to 1000 m above sea level. The area is characterized by a typical subtropical monsoon climate with a mean annual air temperature of 21.4°C. The mean annual precipitation is approximately 1956 mm with a bimodal distribution pattern between April-September and October-March. The average relative humidity is about 80%. The three forests, *Pinus massoniana* forest (PMF), pine and broadleaf mixed forest (PBMF), and monsoon evergreen broadleaf forest (MEBF) represent pioneer, transition and climax (or early, middle and advanced) succession stages, respectively, of the typical monsoon evergreen broadleaved forests in Dinghushan Natural Forest Reserve. The PMF forest, which is approximately 22 ha and was initially planted in 1950s with a single species of *P. massoniana*, occupies the periphery of the reserve. The PBMF forest, which is approximately 557 ha and has developed from an earlier established *P. massoniana* forest, is located between the central area and periphery of the reserve. The MEBF forest, approximately 218 ha, is located in the central area of the reserve and has a stand age of over 400 years old. The characteristics of the experimental sites are shown in Table 1.

**Table 1:** Characteristics of the sampling sites representing a succession gradient.

Forest type	Stand age (year)	Elevation (m)	Slope	Mean air temperature (℃)	Relative humidity (%)	Annual litter fall mass (Mg ha^-1^)
PMF	About 60	130-200	10°-20°	22.7	80	2.53
PBMF	About 110	150-220	10°-25°	20.9	82	7.31
MEBF	About 400	160-230	15°-20°	20.4	87	8.84

*Pinus massoniana* forest (PMF), pine and broadleaf mixed forest (PBMF), and monsoon evergreen broadleaf forest (MEBF), as cited from Ouyang et al. (2005) and Yan et al. (2001)

### Experimental set-up

The study area included 18 experimental plots within the three forest types. Research at these three forest sites has been ongoing since 1979. The results reported here are from a 10 year monitoring campaign from 1999 to 2009. Within each PMF, PBMF and MEBF permanent experimental site, a soil sampling zone with an area of 2000 m^2^ was chosen, based on similarities in soil type, bedrock type of sandstone or shale, elevation, and slope. Six random replicate plots with an area of 6 m × 5 m were established in each of the three forest types.

### Soil sampling

Soil sampling was conducted annually in each of the six study plots per forest type during the month of October (the beginning of the dry season) between 1999 and 2009. In each plot, 9 soil cores down to 20 cm and at 5 m intervals were sampled randomly using a 5 cm diameter soil auger. The soil layer of 0 - 20 cm is where the microbes are most concentrated and the largest amount of organic matter is accumulated. At depths greater than 20 cm, there is a marked decrease in nutrient contents. This sampling approach was chosen in order to minimize the variation at each site. The nine cores were thoroughly mixed for a composite sample. A subsample of this composite sample was immediately weighed using an electronic weighing balance to determine the fresh weight before oven drying at 105°C for 48 hours to obtain dry weight. Water content was determined gravimetrically as the relative change in weight between fresh and dry soil subsamples. Another fresh soil subsample of the composite sample was passed through an 8 mm sieve, and a subsample of about 100 g was air dried, passed through a 0.15 mm diameter sieve and stored in the dark at room temperature prior to chemical total element content analysis and for storage for later use. Another subsample was air dried, passed through a 2 mm diameter sieve, and stored in the dark at room temperature prior to available elements analysis. Results were expressed on an oven-dry weight basis.

### Soil water storage (S)

In addition to the gravimetric soil moisture content determination, soil moisture content was also continuously monitored from 1999 to 2009 using a neutron probe (CPN 503 DR Hydro-probe, CPN International, CA, USA), installed in all the three forest sites. Soil water storage (*S*) was determined as follows:

S(mm)=0.1hmp

$$m\left(\text{\%}\right)=\left(w_{1}-\;w_{2}\right)/w_{2}\text{*1}00\%$$

where *h* is the thickness of soil (20 cm), *m* is the soil water content (%), *p* is the bulk density of soil (g cm^-3^), *w*_*1*_ is wet weight (g), and *w*_*2*_ is dry weight (g). The bulk density of soil (g cm^-3^) is determined as the mass of soil solids (oven-dry) per unit of volume of soil. The volume includes all pore space as well as space occupied by soil particles.

### Soil nutrient content analysis and microbial biomass measurements

Soil properties including soil bulk density, soil pH, soil organic matter (SOM), soil microbial biomass carbon (SMBC), NH_4_^+^-N, available P, K, Fe, Cu, Zn, and B were determined. Soil bulk density was estimated using the core sampling method [21] while soil pH was measured from the air-dried 2 mm-sieved subsamples using a pH electrode with a fresh volume ratio of 1:2 in 0.01 M CaCl_2_. Soil organic matter (SOM) was isolated from the 2 mm-sieved air-dried subsamples according to the methods described by Cambardella and Elliott [22]. NH_4_^+^-N was measured using a microdiffusion method [23]. Available P was analyzed by molybdenum-antimony colorimetry after NH_4_F-HCl extraction. Available B was measured by colorimetric azomethine. Available K, Fe Cu and Zn were analyzed by atomic absorption spectrophotometer after ammonium acetate extraction [23]. Soil microbial biomass carbon (SMBC) was determined with the chloroform fumigation-extraction method [24].

### Statistical analysis

Group means for soil properties measured in the three different forest types were compared using a one-way ANOVA, with the forest types as the fixed effect. First the three types were tested independently to reveal any difference resulting from succession stage. LSD was applied for multiple comparisons (among forest types) when the F test was significant. Data analyses including the correlation analysis were carried out using SPSS version 9.0 (SPSS, Inc., Chicago, Illinois) statistical software. Significance level was set at P<0.05. In all cases, our data passed the normality test hence there was no need for any further data transformation.

## Results

### Soil water storage

**Figure 1:**
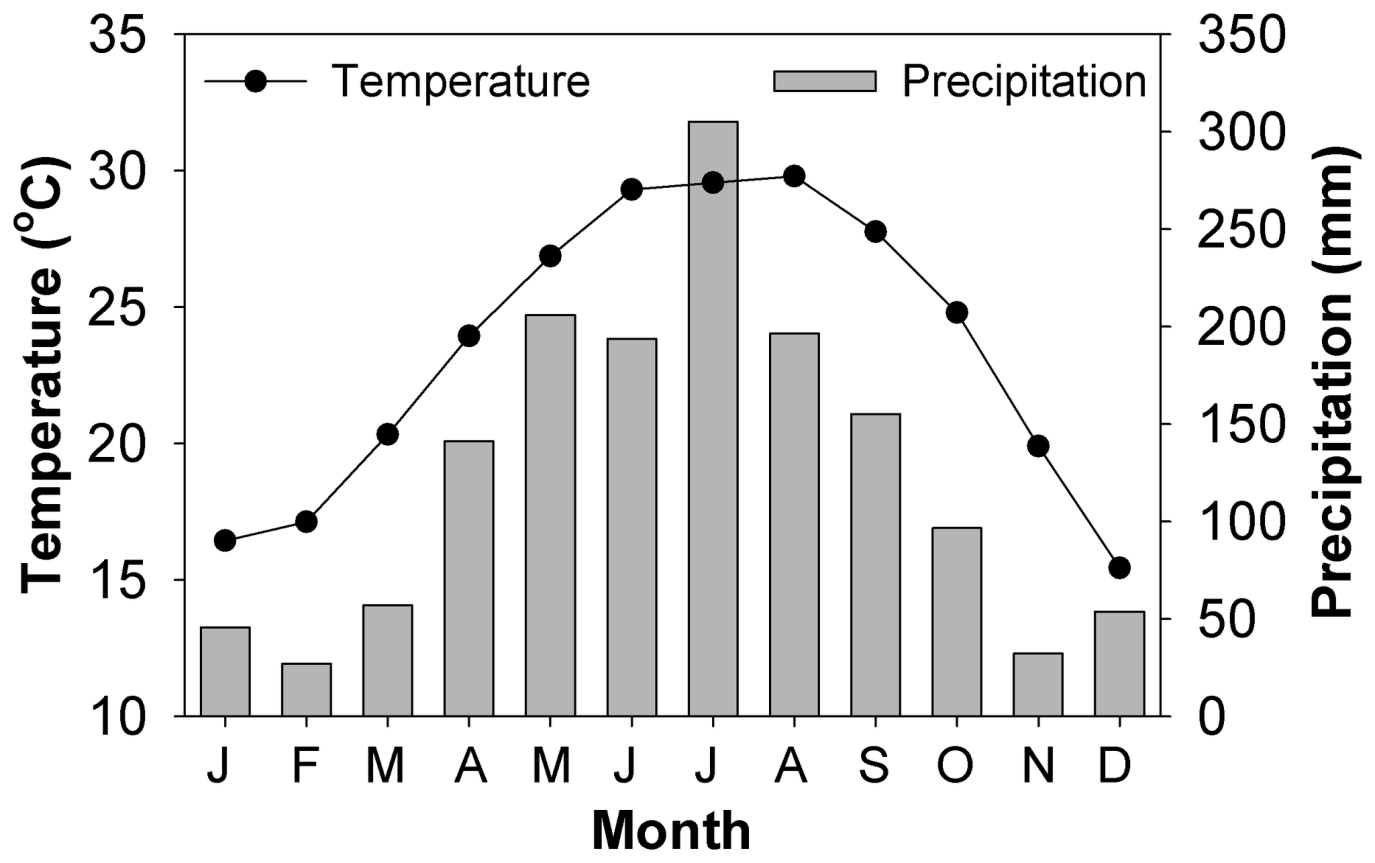
Mean monthly dynamics of temperature and precipitation in the study site from 1999 to 2009.

**Figure 2:**
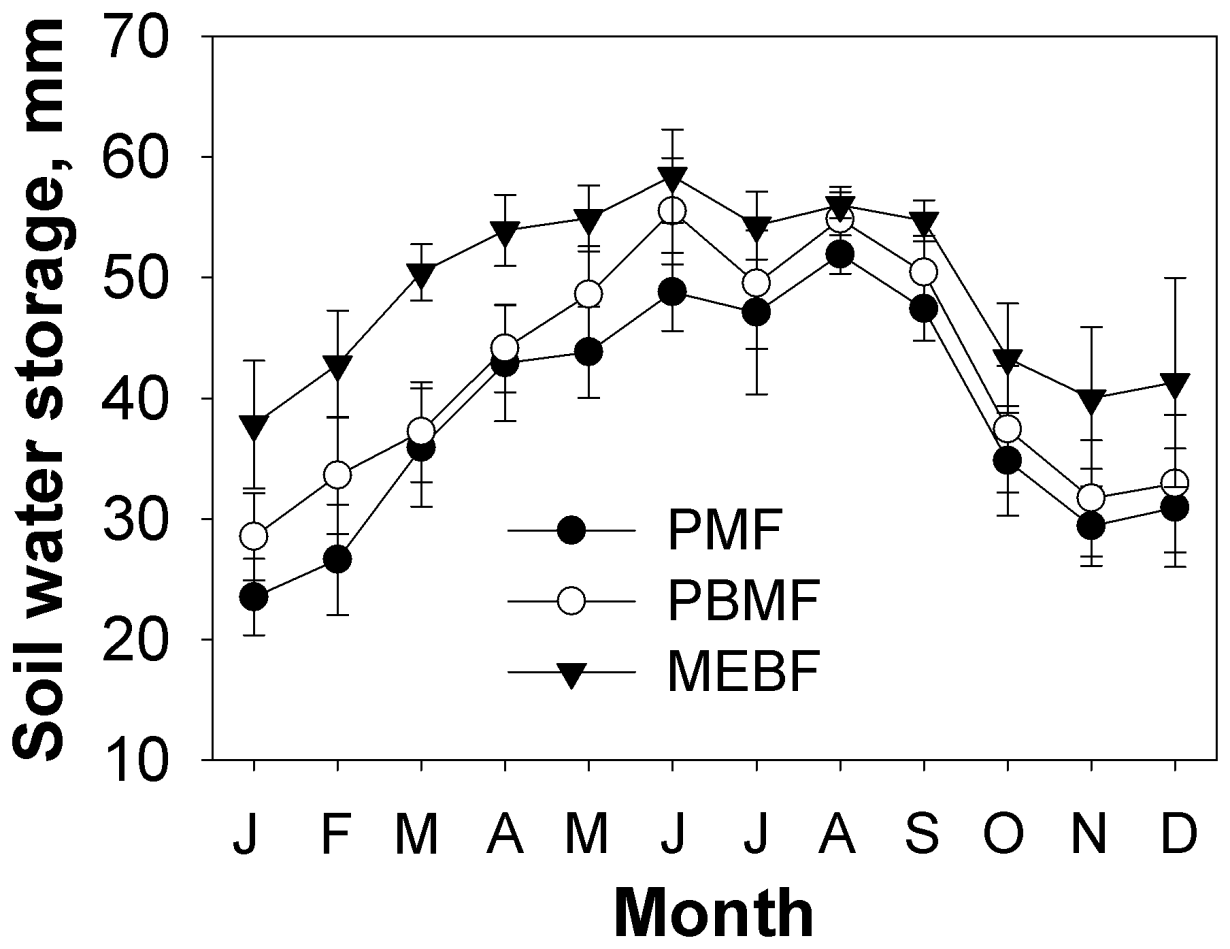
Mean monthly dynamics of soil water storage in the three forest types from 1999 to 2009 (*Pinus massoniana* forest (PMF), pine and broadleaf mixed forest (PBMF), and monsoon evergreen broadleaf forest (MEBF).

Mean monthly temperature and precipitation at the study site from 1999 to 2009 are shown in Figure 1. Approximately 80% of the annual precipitation occurred between April and September. Soil water storage differed significantly among the different forest types (*P*<0.001); the mean monthly soil water storage in MEBF was significantly (*P*<0.001) higher than in the PBMF and PMF (Figure 2). Seasonally, the lowest soil water storage occurred between January and February, while the highest storage was recorded in June and also in August for all the forest types. During the month of July, soil water storage significantly (*P*<0.05) declined despite higher rainfall.

### Soil microbial biomass carbon (SMBC)

**Figure 3:**
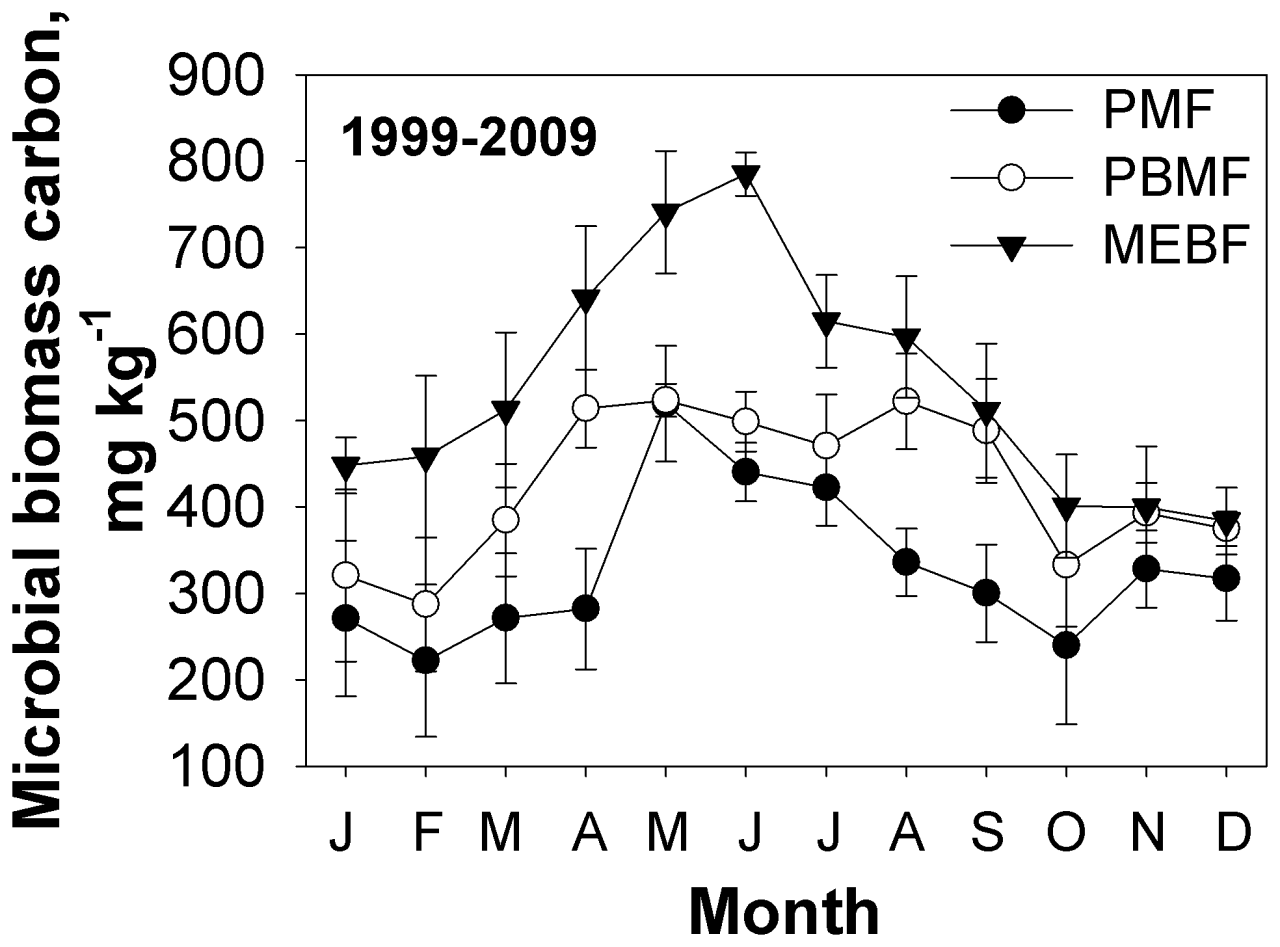
Mean monthly dynamics of soil microbial biomass carbon in the three forest types from 1999 to 2009.

Soil microbial biomass has a major impact on soil properties and processes. Mean monthly soil microbial biomass carbon (SMBC) during the study period (1999-2009) in the three forest types are shown in Figure 3. The highest SMBC was 800 mg kg^-1^ and occurred in the month of June while the lowest value of 200 mg kg^-1^ was recorded during February. The highest SMBC occurred in MEBF, and this was consistent throughout the study period. Differences between MEBF and PBMF and also PMF were significant (P<0.01), but there was no significant difference between PBMF and PMF (*P*>0.05).

### Soil chemical properties

**Figure 4:**
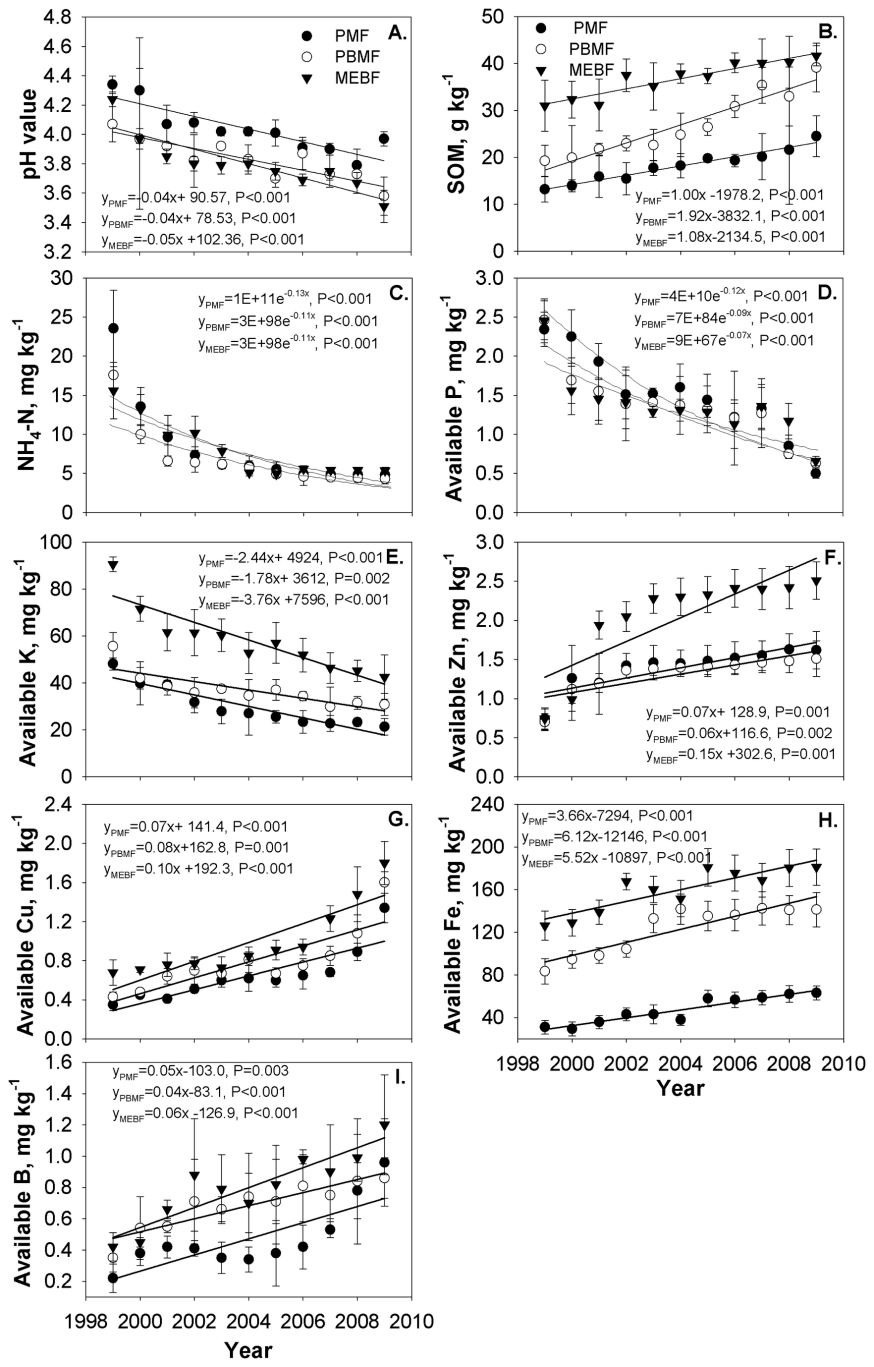
Dynamics of different nutrient contents in the three forest types from 1999 to 2009.

Cations include Al^3+^ (when pH < 5.5), Na^+^, and H^+^. Soil pH of the three different forests ranged between 3.8 and 4.0 throughout the study period (Table 2). Soil pH was negatively and significantly correlated (*P*<0.001，r^2^=0.45) with the succession gradient. There were no significant changes in soil pH between 2003 and 2005, but this was not the case after 2005, with values declining in the PMF. Similar pH decline was observed in PBMF and MEBF between 1999 and 2009 (Figure 4A). Selected soil chemical properties among the different forests varied significantly, with the coefficients of variation ranging between 4% (available P) and 59% (available Fe). Similar variations were also observed for NH_4_^+^-N and available-cations (K, Zn, Cu and B). Among the forests, the most variable parameters were SOM and available Fe (data not shown). 

**Table 2:** Effect of natural succession on selected soil chemical properties (numbers represent mean values±standard error).

Forest Type	pH	SOM (g kg-1)	NH_4_^+^-N (mg kg-1 )	P(m) (mg kg-1 )	K(m) (mg kg-1 )	Zn(m) mg kg-1 )	Cu(m) (mg kg-1 )	Fe(m) (mg kg-1 )	B(m) (mg kg-1 )
PMF	4.04±0.04^a^	19.58±1.02^a^	8.29±1.73^a^	1.50±0.17^a^	30.03±2.63^a^	1.39±0.08^a^	0.65±0.08^a^	47.19±3.69^a^	0.47±0.06^a^
PBMF	3.83±0.04^b^	26.11±2.01^b^	6.84±1.18^a^	1.37±0.14^a^	37.05±2.16^b^	1.31±0.07^b^	0.79±0.10^b^	122.80±6.52^b^	0.68±0.05^b^
MEBF	3.80±0.04^c^	35.85±1.15^c^	8.06±1.11^a^	1.36±0.13^a^	58.52±4.02^c^	2.03±0.18^c^	0.98±0.11^c^	160.0±5.97^c^	0.80±0.07^c^

Different letters within columns indicate significant differences between succession stages forest soils based on LSD (*P* < 0.05). (m)=available cation

### SOM and ammonical nitrogen (NH_4_^+^-N)

SOM content increased with increasing forest age (succession stage) (Figure 4B). The lowest and the highest SOM were 19.58 g kg^-1^ and 35.85 g kg^-1^ and occurred in MEBF and PMF, respectively (Table 2). SOM increased in the three forests during the study period. Differences in SOM concentration among the three forest types were significant (*P*<0.001). SOM in the PBMF exhibited a negative correlation (*P*<0.01, *r* ^2^=0.36) with soil pH. SOM had a positive correlation with NH_4_^+^-N and available Fe（*P*<0.01, *r* ^2^=0.98）in the PMF, and a positive correlation with available B （*P*<0.01, *r* ^2^=0.98）in the MEBF and PBMF.

In contrast to SOM, NH_4_^+^-N decreased in the three forests (Figure 4C). After 2000, NH_4_^+^-N concentrations in the surface runoff were significantly (*P*<0.001) higher in the PMF than in the other two forests. Mean NH_4_^+^-N content over the period was 8.29 mg kg^-1^, 6.84 mg kg^-1^ and 8.06 mg kg^-1^ in PMF, PBMF and MEBF respectively. NH_4_^+^-N content was highest under PMF, suggesting that most conifers generally prefer NH_4_^+^-N when growing under acidic soil. It is important to note here that the PMF site bordered a residential area and the boundary zone was converted into farmland, where higher levels of NH_4_^+^-N content have been registered. However, no significant differences in NH_4_^+^-N were observed along the succession stages (lack of temporal variations). NH_4_^+^-N in soil exhibited a negative correlation with available Cu (*P*<0.05）in the PMF and PBMF. A positive and significant (*P*<0.05) correlation was observed between NH_4_^+^-N and pH in the MEBF. 

### Soil available cations (P, K, Zn, Cu, Fe and B)

Available phosphorus (P) decreased with increasing forest age (succession stage) (Figure 4D). However, there were no significant differences in P between the three forests. The highest P of 2.45 mg kg^-1^ and lowest of 0.5 mg kg^-1^ were recorded in PBMF and PMF during 1999 and 2009 respectively. The highest P loss rate of 78.6% occurred in the PMF. Available P exhibited a positive correlation with pH in PBMF and MEBF types. Available P in soil exhibited a positive correlation with soil pH (*P*<0.01), and with available K and B (*P*<0.01) in PBMF and MEBF, but a negative correlation with available K in PMF (*P*<0.01). 

The highest variation in K in the MEBF was observed between 1999 and 2009, and it ranged from 90.52 mg kg^-1^ to 42.5 mg kg^-1^. Similar but lower variations ranging between 48.14 mg kg^-1^ and 21.38 mg kg^-1^ and 55.64 mg kg^-1^ and 30.81 mg kg^-1^ occurred in the PMF and PBMF respectively (Figure 4E). Available K content and inter-annual trends were different among the forest types. There was a significant correlation between available K with available P. PMF had smaller available K pool than MEBF forest type. 

Soil available zinc (Zn) and copper (Cu) showed an increasing trend with forest age (succession stages) (Figure 4F-G). Significant increases in Zn and Cu concentrations in the forest soils occurred between 1999 and 2009. A similar trend was observed for soil available iron (Fe), showing increasing concentrations in the different forests in the order MEBF>PBMF>PMF (Figure 4H). Furthermore, significant differences in Fe was (*P*<0.05) recorded in different stages of forest succession. Compared to Fe, soil B showed a declining trend in 2003, before increasing from 2005 to 2009 (Figure 4I). There was a positive correlation between B and SOM in PBMF and MEBF, and with available K in PMF (*P*<0.01). 

## Discussion

The results of this study highlight changes in soil properties during the succession of subtropical forests and how they relate to key drivers of forest change such as soil water availability, soil chemical properties, and soil microbial biomass carbon. Our results showed that all these factors impact the pattern of soil change during the succession stages, ultimately leading to positive or negative feedbacks between soil property and forest succession.

### Forest succession and soil water storage

In the series of forest succession, our results showed that the monthly soil water storage capability increased in the three forest types, ranking as MEBF>PMF>PBMF. In general, the changing soil water storage pattern could be explained by annual precipitation pattern, with the soil water storage decreasing in January and February, changes that are associated with reduced amounts of rainfall during this period (i.e. dry season). Despite changes in soil water storage associated with rainfall dynamics, it was significantly evident that water storage capacity of forest soils has an increasing tendency, with forest age (Figure 2). For a similar amount of rainfall, soils in old forest stands, therefore, tend to retain more water than soils under younger forests. Similar findings have been reported in related studies elsewhere [25,26]. The observed decline in soil water storage in July, a period when large amount of monsoon rainfall occurs may be due to frequent storms and heavy rain events, which facilitate rapid runoff and decrease the soil infiltration rate. The monsoon period is also characterized by high radiation levels and windspeeds, factors which favour accelerated water loss from soils due to increased evapotranspiration rates. 

As reported in earlier studies, the water content of litter in the three forest communities differed significantly, in the order MEBF> PBMF> PMF [27]. The evaporation ratio of litter water to free water in MEBF, PBMF and PMF were 78.9%, 82.45% and 91.22%, respectively [27]. Obviously, the canopy architecture of well matured forest type reduced the evaporation loss too. As a result, MEBF soil water storage is the largest.

### Forest succession and soil microbial biomass carbon (SMBC)

Soil microbial biomass carbon (SMBC) was significantly influenced by forest age, date of sampling and soil type [28,29]. In the three forests studied, SMBC ranged from 200 to 800 mg kg^-1^(Figure 3), which is higher than SMBC values of 280-480 mg kg^-1^ reported for African tropical forests [30]. Our values are also higher than values of 380.8 to 568.3 mg kg^-1^ reported in the rubber plantations also in a monsoon-dominated climate [31]. Our SMBC rates are, however, lower than 1080 mg kg^-1^ reported in the temperate forests [32]. SMBC during the dry winter season was less than the wet summer season, which is consistent with the results of Pan et al., 2000 reported for rubber plantations [31]. According to phenological observations for Dinghushan biosphere reserve, most of the plants are in the budding stage from March to May, and then grow vigorously from April to September; the input of fresh organic matter is accompanied by the activation of various rhizosphere microbes. These conditions are conducive to microbial growth and metabolism activity. Hence, soil microbial biomass carbon resulted from a comprehensive abiotic and biotic interaction.

### Forest succession and soil chemical properties

Our results showed that the soils in Dinghushan were predominantly acidic, which can be accounted for by a considerable amount of litter decomposition and high soil nutrients, as well as rapid development of heavy industry in the surrounding area. High acid content in the rainfall of this area resulting from increasing industrial and agricultural activities had been reported in previous studies [33,34]. On the other hand, we tend to think that despite high precipitation, seasonal drought could also explain the phenomenon. In comparison, soil pH was slightly lower in PBMF and MEBF than PMF. Low pH in PBMF and MEBF soil was probably caused by less standing litter fall as a result of the relatively lower rainfall received as well as lower humidity (Table 1). Similar results had been reported in previous studies [35]. Hence, we speculate that soil pH may be decreased under the process of forest succession with the litter fall influencing the pattern except for the environmental abotic driving factors or other biotic driving factors. Of course, such a complicated acidification mechanism deserves further research to understand the process of decomposition of litter fall in MEBF, how the organic acid is produced, where the acid substance comes from, and whether it resulted from organic acid production of microorganisms or hydrogen ions (H^+^) produced by nitrification. Forest types are affected by environmental and geographic conditions, and accordingly so are the soil properties. 

Both SOM and available Fe were rich in the three forests, which may have been caused by the differences in soil conditions and the vegetation community, because during forest succession, litterfall nutrient fluxes increase significantly. The higher inputs of organic matter and nutrients through litterfall positively affect SOM. SOM has been shown to be a good indicator of soil nutrient supply, amelioration of soil properties and prevention of soil erosion [36]. Iron and organic carbon have a strong link either through co-precipitation and/or direct chelation that promote the preservation of organic carbon in the soil. As had been studied earlier in the Brazilian Amazon [37], SOM in lower tropical China was related to soil texture, with its quantity and quality varying with different forest management options. Similar results had been found earlier in SOM under different forest types in Southern China [38]. 

### Forest succession and SOM and NH_4_^+^-N

The pattern for organic matter largely depends on litter abundance and decomposition; and an increase in SOM content of a succession series is mainly caused by decomposition and accumulation of forest litter [39]. Our results showed that SOM content increased with succession stage. The results obtained were consistent with the previous findings that soil organic carbon concentration increased over time within the top 0-20 cm layer in the preserved old-growth forests [12]. This was consistent with our hypothesis. Nitrogen is a major limiting nutrient in most forest ecosystems, especially in the temperate zones [40,41]. Different forest ecosystems have various responses to the N deposition during their succession stages. Old-growth forests are expected to have higher available N than young forests, although during succession, old-growth ecosystems are expected to lose more N than the younger ones [20]. This study confirms earlier research findings suggesting that nitrogen availability increases during primary succession but its transformation differs between primary and secondary succession [42]. Patterns of nitrogen availability varied during succession and that any variability in availability can only be explained by the nature of disturbance [43], as was shown in PMF site which bordered a residential area and farm land. Our study, therefore, suggested that N nitrification has an increasing tendency with advancing forest succession.

### Forest succession and available cations (P, K, Zn, Cu, Fe and B)

Our results showed that available K correlated with available P significantly; and the available K pool of PMF in the primary succession stage was lower as compared to MEBF forest type in the climax succession stage. This was consistent with the results of a study of a tropical wet forest soil in Costa Rica [44]. Plant cover type and its considerable influence on soil properties has been reported in earlier studies [45,46], with lowland tropical forests having less P returned to the soil through litterfall. In stable environments, however, soil nutrient loss is gradual under relatively humid conditions as reported in Hawaiian ecosystems [47]. Differences between soil quality indexes is mainly caused by various biotic and abiotic conditions such as forest soil litter, microbial biomass and their associated activities, and the initial soil properties. In tropical forest ecosystems, soil property is very complex due to having a closer relationship with succession. Our results for this lower subtropical forest are consistent with studies reporting complicated changes of soil properties with forest succession, but long term monitoring should be implemented for a more complete understanding. The pattern for available Zn, Cu, Fe and B shows an increasing tendency in forest succession which is explained by the increase in organic matter that provides more exchangeable sites for cations, or the influence of the pH value, because H ions are released by the decomposition of organic matter and by direct root exudation. All four elements favour leaching of nutrient cations because of competition for the exchange sites. The pattern of the four elements was consistent with our hypothesis. More investigation is necessary for a better understanding of exchangeable Zn, Cu, Fe and B.

### Limitation of the study

Due to the nature of this field study with chronosequence design, it was difficult to find true replications of the forests, as is often the case when very large scale systems are studied. Inferences made in this study should be read with caution considering on the problem of pseudoreplication. Further studies are needed to draw rigorous conclusions regarding on nutrient dynamics in different successional stages of forest.

## Conclusions

In conclusion, the concentrations of SOM, soil microbial biomass carbon, soil water storage, NH_4_^+^-N and available cations (K, Zn, Cu, Fe and B) were all greater in the advanced succession (MEBF). Only available P and pH were higher in the early succession stage (PMF). For the PMF sites, only pH, SOM, and available P and Fe were affected by succession. However, all soil properties were influenced by the increase in time in PBMF and MEBF. The effects of forest succession seemed to vary with differences in soil initial properties and site position. However, no single generalization adequately explained the range of variability encountered in the course of succession. Therefore, further research is needed to unravel the mechanisms behind succession and the relationship between the different physical and chemical factors in tropical forest soils. 
